# Epidemiological characteristics and antimicrobial resistance profiles of clinical bacterial isolates in a tertiary hospital in Zigong, southwestern China, 2023–2025

**DOI:** 10.3389/fcimb.2026.1947772

**Published:** 2026-10-14

**Authors:** Jianhong Yu, Yu Chen, Xianyu You, Suchuan Zhang

**Affiliations:** Department of Clinical Laboratory, Zigong First People’s Hospital, Zigong, China

**Keywords:** antimicrobial resistance, antimicrobial stewardship, carbapenem resistance, clinical isolates, *Haemophilus influenzae*, linezolid non-susceptibility, surveillance

## Abstract

**Introduction:**

Antimicrobial resistance (AMR) varies across hospitals, patient populations, and care settings, limiting the clinical utility of aggregate surveillance data. We characterized bacterial distribution and antimicrobial resistance at a tertiary hospital in Zigong, southwestern China, during 2023–2025, with explicit stratification by age and care setting.

**Methods:**

This retrospective laboratory surveillance study retained the first isolate of each bacterial species per patient per calendar year. We reinterpreted available minimum inhibitory concentrations (MICs) and inhibition-zone diameters using Clinical and Laboratory Standards Institute (CLSI) M100 (35th edition, 2025). Resistance proportions used antimicrobial-specific tested denominators. We estimated temporal associations with logistic regression, modeling calendar year ordinally, using patient-clustered robust standard errors, and adjusting for age, sex, and care setting; we controlled exploratory comparisons using the Benjamini–Hochberg false discovery rate.

**Results:**

From 18,581 laboratory records, 1,615 repeat records were excluded, leaving 16,966 isolates. Isolates were predominantly from general wards (15,478/16,966, 91.2%); 1,330 (7.8%) were ICU/critical-care isolates, and 158 (0.9%) were outpatient isolates. Carbapenem resistance occurred in 70/4,848 *Escherichia coli* (1.4%) and 63/1,769 *Klebsiella pneumoniae* (3.6%). Carbapenem-resistant *Pseudomonas aeruginosa* (CRPA) occurred in 190/1,659 isolates (11.5%), and carbapenem-resistant *Acinetobacter baumannii* (CRAB) in 128/380 isolates (33.7%); ICU/critical-care status was associated with both phenotypes (adjusted OR 2.80 and 4.29, respectively). Phenotypic linezolid non-susceptibility was observed in 60/325 *Enterococcus faecalis* isolates (18.5%; annual range 17.3%–19.0%) without a temporal trend. Among adult *Haemophilus influenzae*, ampicillin resistance increased from 35/50 (70.0%) to 214/243 (88.1%; adjusted OR/year 1.73, 95% CI 1.19–2.50), and amoxicillin/clavulanate resistance increased from 8/50 (16.0%) to 145/243 (59.7%; adjusted OR/year 3.06, 95% CI 2.18–4.30).

**Discussion:**

AMR differed by care setting and age. Carbapenem resistance in *E. coli* and *K. pneumoniae* remained uncommon, whereas CRAB and CRPA were concentrated in ICU/critical-care isolates. The substantial proportion of phenotypic linezolid non-susceptibility in *E. faecalis* warrants confirmation by reference susceptibility testing, and increasing β-lactam resistance among adult *H. influenzae* warrants continued surveillance. These findings support regularly updated institution- and care-setting-specific cumulative antibiograms, with age stratification where isolate numbers are adequate; causal inferences regarding stewardship effects or patient outcomes require linked clinical datasets.

## 1 Introduction

Antimicrobial resistance (AMR) is a major threat to global health and health-system sustainability. Bacterial AMR was directly responsible for an estimated 1.27 million deaths in 2019, and global burden analyses project substantial mortality through 2050 if effective countermeasures are not sustained (Antimicrobial Resistance Collaborators, 2022; GBD 2021 Antimicrobial Resistance Collaborators, 2024). AMR is shaped by antimicrobial exposure, healthcare- and community-associated transmission, infection-prevention practices, population structure, referral pathways, and diagnostic capacity (Okeke et al., 2024). Because these determinants vary within and between health systems, cumulative antimicrobial susceptibility reports should be generated from well-defined local populations and interpreted in their clinical context. CLSI M39 specifically emphasizes standardized methods for cumulative antibiograms used in clinical decision-making and antimicrobial stewardship (Clinical and Laboratory Standards Institute, 2022).

The massive scale of China provides a clear picture of this complexity. Data from the CHINET network continuously show that while Gram-negative pathogens remain the most common, their specific resistance profiles differ wildly depending on the geographic area, the individual facility, the type of clinical specimen, and the patient demographic (Qin et al., 2024; Guo et al., 2025; Ding et al., 2026). Alongside this, nationwide evaluations reveal that China’s overall AMR issue is both severe and constantly shifting (Chen et al., 2025). When looking at carbapenem-resistant Gram-negative bacteria over the last ten years, for instance, there hasn’t been a single, sweeping national pattern; instead, each organism has essentially followed its own unique path (Guo et al., 2026). It is also important to note that datasets gathered from intensive care units or specifically from bloodstream infections focus on highly specific patient profiles. Because of this, trying to compare them directly to general, hospital-wide records is fundamentally flawed (Hu et al., 2022; Chang et al., 2023). Age acts as another crucial factor here, given that the types of bacteria present—and how they resist treatment—shift dramatically when looking across pediatric, adult, and geriatric groups.

Long-term records of antimicrobial resistance from tertiary hospitals outside the major cities in Southwest China are scarce. Zigong, in Sichuan province, presents a unique situation, as its patients come from different demographics and follow different referral paths than those from the major cities of Chengdu and Chongqing. To fill this gap in knowledge, we conducted a three-year retrospective review of laboratory surveillance at Zigong First People’s Hospital. We aimed to determine the types of microorganisms present and their rates of antimicrobial resistance. Additionally, we compared the types and resistance of microorganisms from outpatients, general wards, and intensive care; compared the resistance of microorganisms from pediatric, adult, and older adult patients; and examined changes in the rates of several high-priority antimicrobial resistance phenotypes over the three years. Since our data came only from microbiology logs and not clinical data, we did not investigate the causes of the resistance, such as antimicrobial exposure or stewardship programs.

## 2 Materials and methods

### 2.1 Study design and setting

We conducted a retrospective, laboratory-based surveillance study analyzing clinical bacterial isolates collected from January 2023 to December 2025 at Zigong First People’s Hospital. This facility is a tertiary general hospital equipped with roughly 3,000 beds that serves the Zigong area and its surrounding communities. Non-repetitive isolates served as the primary unit of analysis. Following guidelines from the Clinical and Laboratory Standards Institute (2022), we kept only the initial isolate of any specific bacterial species per patient during a single calendar year (Clinical and Laboratory Standards Institute, 2022). We applied this rule regardless of the specimen’s source or its susceptibility pattern. This meant a single individual might provide multiple different bacterial species in one year or contribute data across multiple different years. We relied on the original laboratory and clinical classifications to exclude common environmental contaminants and typical normal flora. Our research team did not attempt to independently re-evaluate whether an isolate represented a true infection rather than mere colonization. The Ethics Review Committee at Zigong First People’s Hospital granted approval for this research. Since our work relied entirely on anonymized, retrospective microbiological records, the committee waived the need for written informed consent.

Patient ages were divided into three groups: pediatric patients (under 18 years), adults (18 to 64 years), and older adults (65 years and above). We classified the clinical care settings into outpatient departments, general inpatient wards, and intensive care units (ICUs). The ICU category encompassed general, respiratory (RICU), emergency (EICU), neonatal (NICU), and pediatric (PICU) units. Finally, specimen sources were sorted into respiratory samples (such as sputum and bronchoalveolar lavage), urine, blood, abdominal fluid, pus or secretions, and an “other” designation.

### 2.2 Microbiology methods and breakpoint harmonization

Specimen processing, bacterial isolation, and cultivation followed the National Guide to Clinical Laboratory Procedures, 4th edition (Shang et al., 2015). Bacterial identification was performed using the VITEK^®^ 2 Compact automated system and VITEK^®^ MS MALDI-TOF MS (bioMérieux, France). Antimicrobial susceptibility testing (AST) was performed using VITEK^®^ 2 Compact cards (GP67, GP68, and GN13), Kirby–Bauer disk diffusion, and gradient diffusion (E-test) as applicable; fastidious organisms, including *Streptococcus* spp., *H. influenzae*, and *M. catarrhalis*, were tested using the ATB™ system. All *enterococcal* isolates initially reported as non-susceptible to vancomycin or linezolid underwent gradient-diffusion retesting, and the final phenotypic category used in the analysis was based on the confirmatory result. Reference broth microdilution and molecular confirmation were not performed; therefore, linezolid findings are reported as phenotypic non-susceptibility rather than confirmed resistance. β-lactamase positivity in *H. influenzae*, when available in the routine laboratory record, was analyzed as an additional microbiological marker.

To minimize bias from changes in interpretive criteria across study years, available raw MICs and inhibition-zone diameters were reinterpreted using a single breakpoint framework: CLSI M100, 35th edition (2025) (Clinical and Laboratory Standards Institute, 2025). Routine quality-control organisms included *S. aureus* ATCC 25923 and ATCC 29213, *E. faecalis* ATCC 29212, *S. pneumoniae* ATCC 49619, *E. coli* ATCC 25922 and ATCC 35218, *K. pneumoniae* ATCC 700603, *P. aeruginosa* ATCC 27853, and *H. influenzae* ATCC 49247; quality-control results were within acceptable ranges throughout the study period.

### 2.3 Definitions

For the focused resistance analyses, carbapenem-resistant *Enterobacterales* (CRE) were defined as *E. coli* or *K. pneumoniae* isolates resistant to at least one interpretable carbapenem result (ertapenem, imipenem, or meropenem) according to CLSI 2025 breakpoints. For *Proteaceae*, imipenem would not be used for CRE classification because of intrinsically reduced susceptibility; however, the primary CRE analyses in this study focused on *E. coli* and *K. pneumoniae*. Carbapenem-resistant *P. aeruginosa* (CRPA) and carbapenem-resistant *A. baumannii* (CRAB) were defined as resistance to at least one interpretable result for imipenem or meropenem. For *E. faecalis*, the linezolid endpoint comprised isolates classified as non-susceptible by the final phenotypic test. Because reference broth microdilution and molecular confirmation were unavailable, this finding is described throughout as phenotypic linezolid non-susceptibility, without inference regarding a specific resistance mechanism. Isolate-level outcomes are described as proportions rather than prevalence. Methicillin-resistant *Staphylococcus aureus* (MRSA) was defined according to the final *S. aureus* AST interpretation under CLSI 2025 criteria. *H. influenzae* β-lactamase positivity was analyzed as recorded in the final routine laboratory result.

### 2.4 Statistical analysis

Categorical variables are presented as counts and proportions. For each organism–antimicrobial combination, isolates lacking an interpretable result for that antimicrobial were excluded from the denominator; results are therefore reported as resistant isolates/isolates tested (n/N, %). Ninety-five percent confidence intervals (CIs) were calculated for priority resistance estimates. Temporal analyses focused on seven clinically prioritized outcomes: CRE in *E. coli*, CRE in *K. pneumoniae*, CRPA, CRAB, *E. faecalis* phenotypic linezolid non-susceptibility, and ampicillin and amoxicillin/clavulanate resistance in adult *H. influenzae*. β-lactamase positivity in adult *H. influenzae* was analyzed as an additional microbiological marker. Unadjusted temporal trend models were reported for all seven outcomes; multivariable adjusted models were additionally reported for CRPA, CRAB, *E. faecalis* phenotypic linezolid non-susceptibility, and adult *H. influenzae* ampicillin and amoxicillin/clavulanate resistance (**Table 1**).

**Table 1:** Multivariable logistic regression for selected priority resistance outcomes.

Outcome	Factor	Adjusted OR (95% CI)	*P* value	Model N
CRPA	Calendar year (per year)	1.20 (0.97-1.48)	0.088	1659
CRPA	ICUs vs general ward	2.80 (1.92-4.07)	<0.001	1659
CRAB	Calendar year (per year)	0.86 (0.64-1.15)	0.301	380
CRAB	ICUs vs general ward	4.29 (2.63-6.99)	<0.001	380
*E. faecalis* linezolid non-susceptibility	Calendar year (per year)	1.01 (0.72–1.42)	0.954	325
*E. faecalis* linezolid non-susceptibility	ICUs vs general ward	2.05 (0.77–5.45)	0.150	325
Adult *H. influenzae* ampicillin resistance	Calendar year (per year)	1.73 (1.19-2.50)	0.004	448
Adult *H. influenzae* ampicillin resistance	ICUs vs general ward	0.86 (0.49-1.51)	0.594	448
Adult *H. influenzae* amoxicillin/clavulanate resistance	Calendar year (per year)	3.06 (2.18-4.30)	<0.001	448
Adult *H. influenzae* amoxicillin/clavulanate resistance	ICUs vs general ward	0.86 (0.54-1.37)	0.530	448

Care setting was modeled as three levels (general ward as reference). Outpatient estimates are not shown because sparse subgroup sizes and zero events made them unstable; interpretation focuses on ICU versus general ward. Models included calendar year, age, sex, and care setting.

Temporal trends were assessed using binomial logistic regression with calendar year coded ordinally (2023 = 0, 2024 = 1, 2025 = 2). Effect estimates are reported as odds ratios (ORs) per calendar-year increase with 95% CIs and *P* values for trend. Because the same patient could contribute different species and, rarely, observations in more than one calendar year, regression models used patient-clustered robust standard errors. The adjusted models reported in **Table 1** included calendar year, age, sex, and care setting. For adult *H. influenzae*, specimen type was not included because nearly all isolates were respiratory, resulting in sparse or non-estimable categories. For the *H. influenzae* temporal analyses, “adult isolates” denotes all isolates from patients aged ≥18 years, combining the 18–64-year and ≥65-year strata.

Exploratory organism–antimicrobial comparisons were corrected using the Benjamini–Hochberg false discovery rate (FDR). Two-sided *P* < 0.05 was considered statistically significant for the clinically prioritized analyses. For exploratory comparisons, q < 0.05 identified FDR-significant signals; all exploratory findings were considered hypothesis-generating. Analyses were performed using WHONET-derived data and Python/statsmodels.

### 2.5 Antimicrobial stewardship context

Zigong First People’s Hospital implements antimicrobial-use governance practices, including prescription review, antimicrobial-use management, and microbiology-based resistance surveillance and feedback. Such practices are common in Chinese tertiary hospitals as well (Zhou and Ma, 2019). However, the microbiology dataset used for the current study did not include data regarding antimicrobial exposure at the patient level, days of therapy or defined daily doses, prescriber-level interventions, or the dates or details of any stewardship-policy changes during 2023–2025. Therefore, the study did not model the exposure or effectiveness of antimicrobial stewardship practices, nor did it attribute any temporal changes in antimicrobial resistance to such practices.

## 3 Results

### 3.1 Study cohort and isolate distribution

From 18,581 records across the 12 quarterly microbiology datasets, 1,615 repeats were removed under the patient–species–calendar-year rule. That left 16,966 non-repetitive isolate-level observations from 15,095 unique patients (**Figure 1**). Yearly isolate counts were 5,081 in 2023, 5,536 in 2024, and 6,349 in 2025. Gram-negative isolates made up 12,870 (75.9%) of the total isolates, compared with 4,096 (24.1%) Gram-positive isolates. In terms of age, 4,104 (24.2%) isolates originated from patients younger than 18 years, 5,311 (31.3%) from patients between 18 and 64 years of age, and 7,551 (44.5%) from patients 65 years or older. Only 158 (0.9%) isolates originated from outpatients; general wards contributed 15,478 (91.2%) isolates, and ICUs contributed 1,330 (7.8%). The leading source of isolates was respiratory specimens (7,384; 43.5%), followed by urine (3,612; 21.3%), abdominal fluid (2,216; 13.1%), pus/secretion (2,098; 12.4%), and blood (1,316; 7.8%) (**Table 2**).

**Figure 1:**
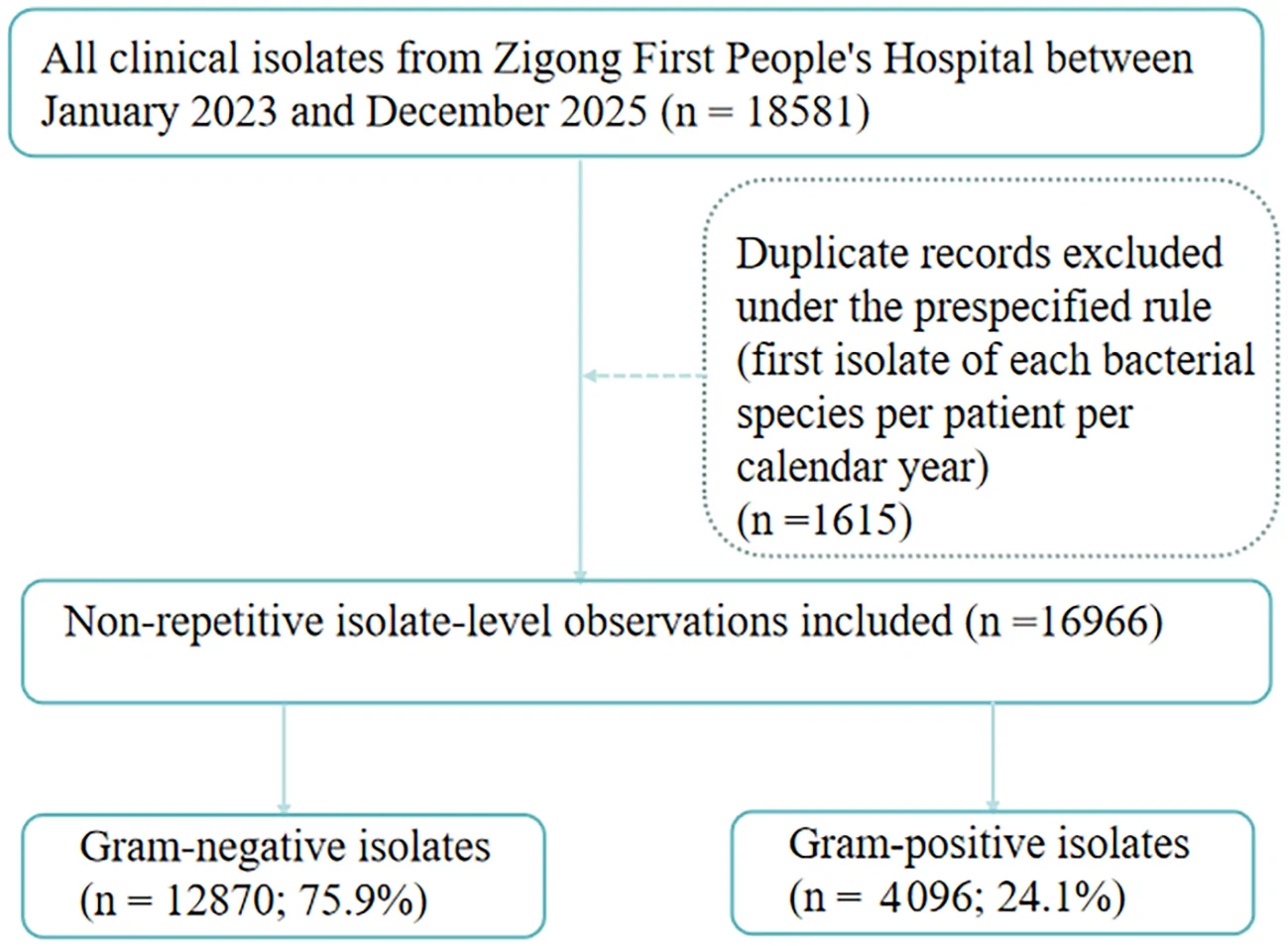
Flow diagram of isolate selection.

**Table 2:** Baseline characteristics of included isolate-level observations by year.

Characteristic	2023	2024	2025	Overall
Total isolates	5081	5536	6349	16966
Male	2625 (51.7%)	2910 (52.6%)	3300 (52.0%)	8835 (52.1%)
Female	2456 (48.3%)	2626 (47.4%)	3049 (48.0%)	8131 (47.9%)
Age <18 years	1255 (24.7%)	1351 (24.4%)	1498 (23.6%)	4104 (24.2%)
Age 18–64 years	1615 (31.8%)	1788 (32.3%)	1908 (30.1%)	5311 (31.3%)
Age ≥65 years	2211 (43.5%)	2397 (43.3%)	2943 (46.4%)	7551 (44.5%)
Outpatient	43 (0.8%)	33 (0.6%)	82 (1.3%)	158 (0.9%)
General ward	4618 (90.9%)	5053 (91.3%)	5807 (91.5%)	15478 (91.2%)
ICUs	420 (8.3%)	450 (8.1%)	460 (7.2%)	1330 (7.8%)
Respiratory specimen	2056 (40.5%)	2391 (43.2%)	2937 (46.3%)	7384 (43.5%)
Urine	1192 (23.5%)	1141 (20.6%)	1279 (20.1%)	3612 (21.3%)
Abdominal fluid	695 (13.7%)	718 (13.0%)	803 (12.6%)	2216 (13.1%)
Pus/secretion	641 (12.6%)	708 (12.8%)	749 (11.8%)	2098 (12.4%)
Blood	412 (8.1%)	428 (7.7%)	476 (7.5%)	1316 (7.8%)

The most frequently recovered organisms were *E. coli* (4,848; 28.6%), followed by *K. pneumoniae* (1,769; 10.4%), *P. aeruginosa* (1,659; 9.8%), *H. influenzae* (1,567; 9.2%), *S. aureus* (1,434; 8.5%), *M. catarrhalis* (907; 5.3%), *S. pneumoniae* (904; 5.3%), *S. agalactiae* (450; 2.7%), *E. faecium* (409; 2.4%), and *A. baumannii* (380; 2.2%) (**Table 3**). Age and care setting had a clear effect on the spectrum: pediatric isolates were enriched for *H. influenzae*, *M. catarrhalis*, and *S. pneumoniae*, whereas *P. aeruginosa*, *K. pneumoniae*, *A. baumannii*, and *enterococci* were more prominent in older-adult and/or ICU isolates (**Table 4**). The distribution of the leading organisms also differed across major specimen groups (**Supplementary Table S1**).

**Table 3:** Most frequently recovered organisms by year.

Organism	2023	2024	2025	Overall
*E. coli*	1590 (31.3%)	1544 (27.9%)	1714 (27.0%)	4848 (28.6%)
*K. pneumoniae*	496 (9.8%)	592 (10.7%)	681 (10.7%)	1769 (10.4%)
*P. aeruginosa*	439 (8.6%)	547 (9.9%)	673 (10.6%)	1659 (9.8%)
*H. influenzae*	262 (5.2%)	594 (10.7%)	711 (11.2%)	1567 (9.2%)
*S. aureus*	466 (9.2%)	458 (8.3%)	510 (8.0%)	1434 (8.5%)
*M. catarrhalis*	269 (5.3%)	264 (4.8%)	374 (5.9%)	907 (5.3%)
*S. pneumoniae*	372 (7.3%)	249 (4.5%)	283 (4.5%)	904 (5.3%)
*S. agalactiae*	130 (2.6%)	179 (3.2%)	141 (2.2%)	450 (2.7%)
*E. faecium*	125 (2.5%)	124 (2.2%)	160 (2.5%)	409 (2.4%)
*A. baumannii*	140 (2.8%)	121 (2.2%)	119 (1.9%)	380 (2.2%)

**Table 4:** Distribution of major organisms by care setting and age group. .

Organism	Outpatient	General ward	ICUs	<18 years	18–64 years	≥65 years
*E. coli*	64 (1.3%)	4548 (93.8%)	236 (4.9%)	575 (11.9%)	2024 (41.7%)	2249 (46.4%)
*K. pneumoniae*	12 (0.7%)	1512 (85.5%)	245 (13.8%)	97 (5.5%)	587 (33.2%)	1085 (61.3%)
*P. aeruginosa*	11 (0.7%)	1500 (90.4%)	148 (8.9%)	88 (5.3%)	488 (29.4%)	1083 (65.3%)
*H. influenzae*	3 (0.2%)	1501 (95.8%)	63 (4.0%)	1119 (71.4%)	96 (6.1%)	352 (22.5%)
*S. aureus*	27 (1.9%)	1329 (92.7%)	78 (5.4%)	505 (35.2%)	533 (37.2%)	396 (27.6%)
*M. catarrhalis*	1 (0.1%)	886 (97.7%)	20 (2.2%)	768 (84.7%)	48 (5.3%)	91 (10.0%)
*S. pneumoniae*	2 (0.2%)	864 (95.6%)	38 (4.2%)	696 (77.0%)	63 (7.0%)	145 (16.0%)
*S. agalactiae*	1 (0.2%)	447 (99.3%)	2 (0.4%)	3 (0.7%)	389 (86.4%)	58 (12.9%)
*E. faecium*	1 (0.2%)	320 (78.2%)	88 (21.5%)	25 (6.1%)	96 (23.5%)	288 (70.4%)
*A. baumannii*	0 (0.0%)	284 (74.7%)	96 (25.3%)	9 (2.4%)	80 (21.0%)	291 (76.6%)

Percentages are row percentages within each organism.

### 3.2 Priority resistance phenotypes by care setting and age

Priority resistance phenotypes varied across care settings (**Table 5**). CRPA and CRAB proportions were markedly higher in ICUs than in general wards (27.7% vs. 9.9% and 68.8% vs. 21.8%, respectively). In contrast, CRE proportions remained low (≤4.1%) in both general wards and ICUs. Outpatient subgroup sizes for most priority organisms were small, producing imprecise estimates with wide confidence intervals; these outpatient resistance percentages should therefore not be interpreted as a representative community antibiogram.

**Table 5:** Priority resistance phenotypes by care setting.

Organism	Outcome	Outpatient	General ward	ICUs
*E. coli*	CRE	1/64 (1.6%; 0.3-8.3)	64/4548 (1.4%; 1.1-1.8)	5/236 (2.1%; 0.9-4.9)
*K. pneumoniae*	CRE	0/12 (0.0%; 0.0-24.2)	53/1512 (3.5%; 2.7-4.6)	10/245 (4.1%; 2.1-7.6)
*P. aeruginosa*	CRPA	0/11 (0.0%; 0.0-25.9)	149/1500 (9.9%; 8.5-11.6)	41/148 (27.7%; 21.1-35.4)
*A. baumannii*	CRAB	0/0 (proportion and 95% CI not estimable)	62/284 (21.8%; 17.4-27.0)	66/96 (68.8%; 58.9-77.1)
*E. faecalis*	Linezolid non-susceptibility	0/4 (0.0%; 0.0-60.2)	54/296 (18.2%; 14.3-23.0)	6/25 (24.0%; 11.5-43.4)
*H. influenzae*	Ampicillin resistance	1/3 (33.3%; 6.1-79.2)	1291/1501 (86.0%; 84.1-87.7)	48/63 (76.2%; 64.4-85.0)
*H. influenzae*	Amoxicillin/clavulanate resistance	1/3 (33.3%; 6.1-79.2)	650/1501 (43.3%; 40.8-45.8)	20/63 (31.7%; 21.6-44.0)
*S. aureus*	MRSA	1/27 (3.7%; 0.7-18.3)	320/1329 (24.1%; 21.8-26.4)	19/78 (24.4%; 16.2-34.9)

Values are n/N (%; 95% CI).

Age-stratified analyses are shown in **Table 6**. Carbapenem resistance in *E. coli* and *K. pneumoniae* remained low across age strata, whereas CRPA and CRAB were more frequent among adult and older-adult isolates than among pediatric isolates. Phenotypic linezolid non-susceptibility in *E. faecalis* was observed in each age group, although the pediatric denominator was small. *H. influenzae* β-lactam resistance also differed by age group. MRSA proportions were broadly similar across pediatric and adult groups, with a numerically higher proportion among older adults.

**Table 6:** Priority resistance phenotypes by age group.

Organism	Outcome	<18 years	18–64 years	≥65 years
*E. coli*	CRE	1/575 (0.2%; 0.0-1.0)	32/2024 (1.6%; 1.1-2.2)	37/2249 (1.6%; 1.2-2.3)
*K. pneumoniae*	CRE	1/97 (1.0%; 0.2-5.6)	24/587 (4.1%; 2.8-6.0)	38/1085 (3.5%; 2.6-4.8)
*P. aeruginosa*	CRPA	1/88 (1.1%; 0.2-6.2)	82/488 (16.8%; 13.7-20.4)	107/1083 (9.9%; 8.2-11.8)
*A. baumannii*	CRAB	1/9 (11.1%; 2.0-43.5)	26/80 (32.5%; 23.2-43.4)	101/291 (34.7%; 29.5-40.3)
*E. faecalis*	Linezolid non-susceptibility	3/17 (17.6%; 6.2-41.0)	24/120 (20.0%; 13.8-28.0)	33/188 (17.6%; 12.8-23.6)
*H. influenzae*	Ampicillin resistance	962/1119 (86.0%; 83.8-87.9)	83/96 (86.5%; 78.2-92.0)	295/352 (83.8%; 79.6-87.3)
*H. influenzae*	Amoxicillin/clavulanate resistance	463/1119 (41.4%; 38.5-44.3)	69/96 (71.9%; 62.2-79.9)	139/352 (39.5%; 34.5-44.7)
*S. aureus*	MRSA	118/505 (23.4%; 19.9-27.2)	113/533 (21.2%; 17.9-25.0)	109/396 (27.5%; 23.4-32.1)

Values are n/N (%; 95% CI).

### 3.3 Temporal trends in priority resistance phenotypes

Temporal-trend results are summarized in **Table 7**; **Figure 2**. *E. coli* CRE remained stable, from 21/1,590 (1.3%) in 2023 to 29/1,714 (1.7%) in 2025 (OR/year 1.14, 95% CI 0.86–1.52; *P* = 0.360). *K. pneumoniae* CRE likewise showed no monotonic trend (4.4%, 2.7%, and 3.7%; OR/year 0.91, 95% CI 0.68–1.22; *P* = 0.545). CRPA increased from 38/439 (8.7%) to 91/673 (13.5%) in the unadjusted trend model (OR/year 1.28, 95% CI 1.05–1.55; *P* = 0.013), whereas CRAB declined numerically from 54/140 (38.6%) to 35/119 (29.4%) without a statistically significant trend (OR/year 0.81, 95% CI 0.63–1.05; *P* = 0.119).

**Table 7:** Temporal trends in clinically prioritized resistance outcomes.

Outcome	2023	2024	2025	Absolute change (pp)	OR/year (95% CI)	*P* trend
*E. coli*: CRE	21/1590 (1.3%; 0.9-2.0)	20/1544 (1.3%; 0.8-2.0)	29/1714 (1.7%; 1.2-2.4)	0.4	1.14 (0.86-1.52)	0.360
*K. pneumoniae*: CRE	22/496 (4.4%; 2.9-6.6)	16/592 (2.7%; 1.7-4.3)	25/681 (3.7%; 2.5-5.4)	-0.7	0.91 (0.68-1.22)	0.545
*P. aeruginosa*: CRPA	38/439 (8.7%; 6.4-11.7)	61/547 (11.2%; 8.8-14.1)	91/673 (13.5%; 11.1-16.3)	4.8	1.28 (1.05-1.55)	0.013
*A. baumannii*: CRAB	54/140 (38.6%; 30.9-46.8)	39/121 (32.2%; 24.0-41.0)	35/119 (29.4%; 22.0-38.1)	-9.2	0.81 (0.63-1.05)	0.119
*E. faecalis*: Linezolid non-susceptibility	17/98 (17.3%; 11.1-26.0)	19/100 (19.0%; 12.5-27.8)	24/127 (18.9%; 13.0-26.6)	1.6	1.05 (0.75-1.48)	0.777
*H. influenzae* (≥18 years): Ampicillin resistance	35/50 (70.0%; 56.2-80.9)	129/155 (83.2%; 76.6-88.3)	214/243 (88.1%; 83.4-91.6)	18.1	1.73 (1.22-2.45)	0.002
*H. influenzae* (≥18 years): Amoxicillin/clavulanate resistance	8/50 (16.0%; 8.3-28.5)	55/155 (35.5%; 28.4-43.3)	145/243 (59.7%; 53.4-65.6)	43.7	2.78 (2.03-3.83)	<0.001
*H. influenzae* (≥18 years): β-lactamase positivity	35/50 (70.0%; 56.2-80.9)	119/155 (76.8%; 69.5-82.7)	206/243 (84.8%; 79.7-88.7)	14.8	1.55 (1.10-2.18)	0.012

**Figure 2:**
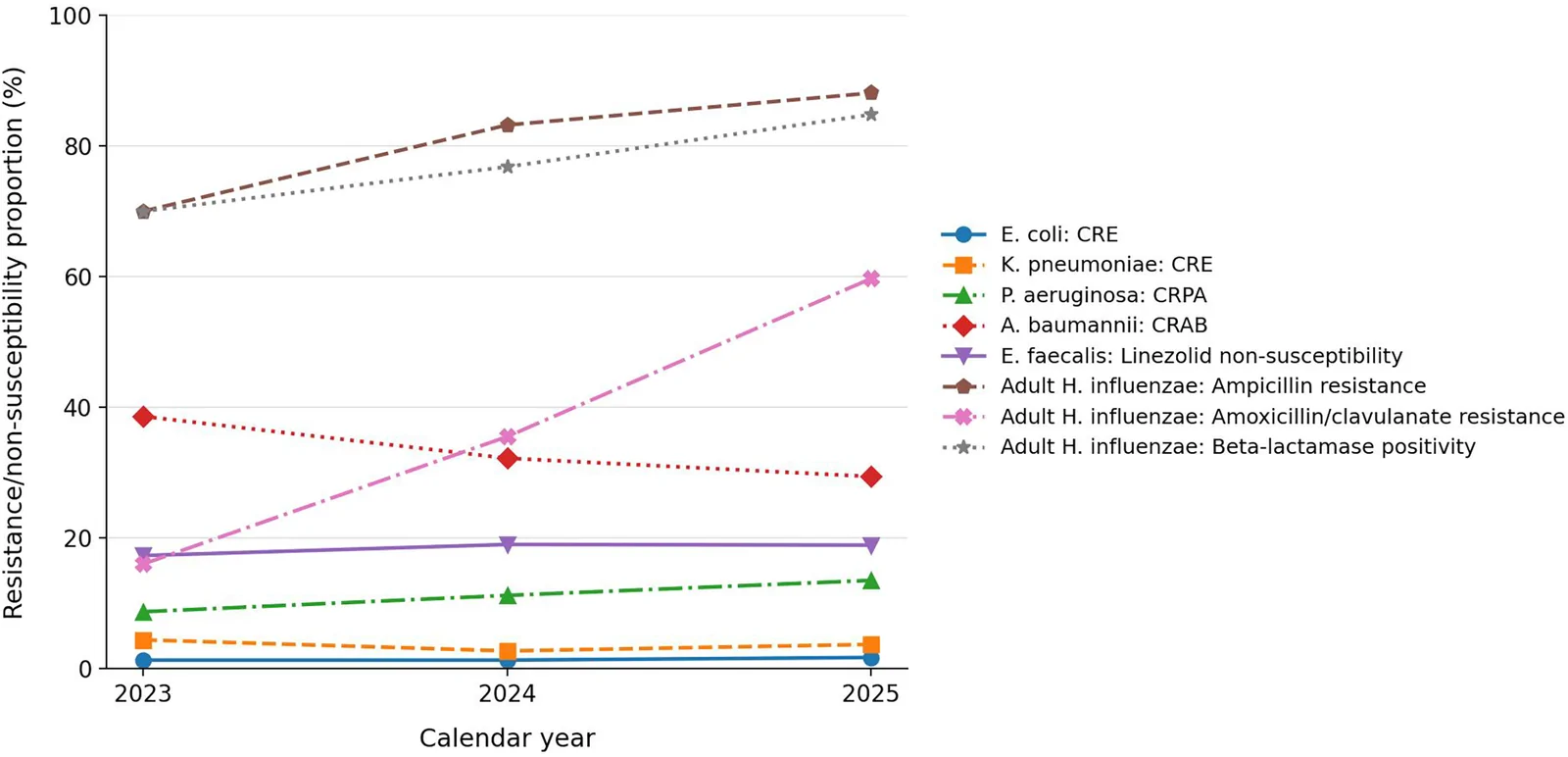
Temporal changes in selected priority resistance outcomes, 2023–2025.

Phenotypic linezolid non-susceptibility in *E. faecalis* was stable across study years: 17/98 (17.3%) in 2023, 19/100 (19.0%) in 2024, and 24/127 (18.9%) in 2025 (OR/year 1.05, 95% CI 0.75–1.48; *P* = 0.777). Among adult *H. influenzae*, ampicillin resistance increased from 35/50 (70.0%) to 214/243 (88.1%) (absolute change +18.1 percentage points; OR/year 1.73, 95% CI 1.22–2.45; *P* = 0.002). Amoxicillin/clavulanate resistance increased from 8/50 (16.0%) to 145/243 (59.7%) (absolute change +43.7 percentage points; OR/year 2.78, 95% CI 2.03–3.83; *P* < 0.001). β-lactamase positivity also increased from 35/50 (70.0%) to 206/243 (84.8%) (OR/year 1.55, 95% CI 1.10–2.18; *P* = 0.012).

### 3.4 Adjusted analyses and patient-level non-independence

Patient-clustered multivariable logistic models are shown in **Table 1**. After adjustment for measured covariates, ICU/critical-care status remained associated with CRPA (adjusted OR 2.80, 95% CI 1.92–4.07; *P* < 0.001) and CRAB (adjusted OR 4.29, 95% CI 2.63–6.99; *P* < 0.001) compared with general-ward care. After adjustment for measured case-mix variables, calendar year was not independently associated with CRPA, CRAB, or *E. faecalis* phenotypic linezolid non-susceptibility; thus, the unadjusted CRPA trend was attenuated after adjustment. In contrast, the calendar-year association persisted for adult *H. influenzae* ampicillin resistance (adjusted OR/year 1.73, 95% CI 1.19–2.50; *P* = 0.004) and amoxicillin/clavulanate resistance (adjusted OR/year 3.06, 95% CI 2.18–4.30; *P* < 0.001). These models included calendar year, age, sex, and care setting; adult *H. influenzae* specimen type was not included because almost all isolates were respiratory.

### 3.5 MIC distributions for selected clinically important phenotypes

MIC distributions provide additional phenotypic context for these priority outcomes (**Table 8**). The MIC_50_ and MIC_90_ were calculated from the numeric MIC results available for each organism/drug combination and are presented descriptively below. For *E. faecalis* and linezolid, the MIC_50_ was 2 mg/L in each of the years studied. The MIC_90_ was 8 mg/L, and the numeric MIC range extended to 16 mg/L in 2023–2024 and 8 mg/L in 2025. For *H. influenzae* from adult patients, the ampicillin MIC_50_ increased from 16 mg/L in 2023 to 64 mg/L in 2024–2025. The amoxicillin/clavulanate MIC_50_ increased from 4 mg/L to 8 mg/L by 2025, and the upper observed MIC increased to 64 mg/L. These descriptive MIC summaries provide phenotypic context but do not identify resistance mechanisms in the organisms.

**Table 8:** MIC distribution of selected organism–antimicrobial combinations.

Organism	Antimicrobial	Year	MIC available (n)	MIC_50_ (mg/L)	MIC_90_ (mg/L)	Observed numeric range (mg/L)
*E. faecalis*	Linezolid	2023	91	2.0	8.0	1-16
*E. faecalis*	Linezolid	2024	98	2.0	8.0	1-16
*E. faecalis*	Linezolid	2025	119	2.0	8.0	1-8
*E. coli*	Ertapenem	2023	1590	0.5	0.5	0.5-8
*E. coli*	Ertapenem	2024	1544	0.5	0.5	0.5-8
*E. coli*	Ertapenem	2025	1714	0.5	0.5	0.5-8
*K. pneumoniae*	Ertapenem	2023	493	0.5	0.5	0.5-8
*K. pneumoniae*	Ertapenem	2024	589	0.5	0.5	0.5-8
*K. pneumoniae*	Ertapenem	2025	679	0.5	0.5	0.5-8
*P. aeruginosa*	Imipenem	2023	390	2.0	4.0	1-16
*P. aeruginosa*	Imipenem	2024	435	2.0	8.0	1-16
*P. aeruginosa*	Imipenem	2025	544	2.0	4.0	1-16
*A. baumannii*	Imipenem	2023	140	1.0	16.0	1-16
*A. baumannii*	Imipenem	2024	120	1.0	16.0	1-16
*A. baumannii*	Imipenem	2025	119	1.0	16.0	1-16
*H. influenzae* (≥18 years)	Ampicillin	2023	50	16.0	64.0	0.25-64
*H. influenzae* (≥18 years)	Ampicillin	2024	155	64.0	64.0	0.25-64
*H. influenzae* (≥18 years)	Ampicillin	2025	243	64.0	64.0	0.25-64
*H. influenzae* (≥18 years)	Amoxicillin/clavulanate	2023	50	4.0	8.0	4-8
*H. influenzae* (≥18 years)	Amoxicillin/clavulanate	2024	155	4.0	8.0	2-16
*H. influenzae* (≥18 years)	Amoxicillin/clavulanate	2025	243	8.0	16.0	2-64

### 3.6 Detailed antimicrobial resistance profiles

Detailed antimicrobial-specific results are provided in **Supplementary Tables S2**, **S3**. Each cell reports resistant isolates among isolates with interpretable AST results. Exploratory temporal *P* values were adjusted using the Benjamini–Hochberg FDR procedure. In addition to the clinically prioritized outcomes, FDR-significant exploratory signals included decreasing *P. aeruginosa* aztreonam resistance and decreasing *A. baumannii* levofloxacin resistance. Because these outcomes were exploratory and secular changes in AST platform/card utilization could not be fully reconstructed, they should be considered hypothesis-generating rather than definitive temporal effects.

## 4 Discussion

This three-year hospital-wide surveillance study provides a detailed local susceptibility profile while explicitly addressing care setting, age, antimicrobial-specific denominators, patient-level correlation, effect sizes, MIC distributions, and multiplicity. Four findings are particularly relevant. First, carbapenem resistance remained uncommon in *E. coli* and *K. pneumoniae*. Second, carbapenem resistance among non-fermenters was concentrated in ICU/critical-care isolates, particularly *A. baumannii*. Third, a substantial proportion of *E. faecalis* isolates showed phenotypic linezolid non-susceptibility, although no temporal increase was detected. Fourth, adult *H. influenzae* showed a marked increase in β-lactam resistance, particularly to amoxicillin/clavulanate, and the calendar-year association persisted after adjustment for measured case mix.

The organism spectrum was strongly shaped by the sampling frame. Gram-negative bacteria accounted for 75.9% of isolates, respiratory specimens for 43.5%, and 24.2% of isolates came from patients aged <18 years. These features help explain the prominence of *H. influenzae*, *S. pneumoniae*, and *M. catarrhalis*. CHINET 2025 similarly reported 71.5% Gram-negative isolates, 42.8% respiratory specimens, and 22.4% pediatric isolates, whereas its outpatient contribution was 9.3% compared with only 0.9% in the present cohort (Ding et al., 2026). Accordingly, our overall estimates primarily describe inpatient microbiology and should not be interpreted as a community antibiogram. This contrast also illustrates why institution-, care-setting-, and age-stratified data can be more clinically informative than national aggregate percentages alone.

Among Enterobacterales, carbapenem resistance in *E. coli* and *K. pneumoniae* remained stable and low across 2023–2025. The broader profiles also showed organism-specific patterns: *E. coli* maintained substantial ceftriaxone resistance (40.4%–42.4%), fluoroquinolone resistance around one-half or higher, and trimethoprim/sulfamethoxazole resistance near one-half, whereas *K. pneumoniae* showed lower resistance to these agents in this hospital (**Supplementary Table S2**). By comparison, CHINET 2025 reported *E. coli* resistance of 2.4%/2.6% to imipenem/meropenem and K. pneumoniae resistance of 21.9%/21.0%, together with higher national ceftriaxone and fluoroquinolone resistance (Ding et al., 2026). Regional multicenter and national longitudinal analyses further demonstrate substantial geographic and temporal heterogeneity in CRE and other carbapenem-resistant Gram-negative bacteria (Guo et al., 2024, Guo et al., 2026). Differences in referral intensity, patient risk profile, antimicrobial exposure, invasive procedures, local clonal structure, carbapenemase distribution, and testing practices are plausible explanations, but most were not measured here and therefore cannot be assigned causal roles.

The findings regarding non-fermenters further demonstrate the importance of care-setting stratification in studying antibiotic resistance. The non-fermenters in this study were primarily *P. aeruginosa* and *A. baumannii*, with overall carbapenem resistance rates of 11.5% and 33.7%, respectively. While carbapenem resistance in *P. aeruginosa* increased over the study period, this trend was no longer statistically significant after accounting for case mix. In contrast, ICU/critical care status was strongly associated with carbapenem resistance in both *P. aeruginosa* (adjusted OR 2.80) and *A. baumannii* (adjusted OR 4.29). These findings align with previous studies demonstrating a significant burden of carbapenem-resistant Gram-negative bacteria in critical care settings in China (Chang et al., 2023; Hou et al., 2025). In exploratory analyses of other antibiotic resistances in these non-fermenting organisms, aztreonam resistance in *P. aeruginosa* and levofloxacin resistance in *A. baumannii* decreased after correcting for false discovery rate. Other resistances in *A. baumannii* did not meet the inferential threshold for significance. According to the CHINET 2025 report, 22.6% and 18.8% of *P. aeruginosa* isolates were resistant to imipenem and meropenem, respectively, while 72.0% and 71.9% of *A. baumannii* isolates demonstrated resistance (Ding et al., 2026). Studies focused on ICU settings in southwestern China and genomic studies of carbapenem-resistant *A. baumannii* in Chinese ICUs have highlighted the importance of healthcare setting and clonal effects on resistance (Chang et al., 2023; Liu et al., 2022, Liu et al., 2024), while studies of healthcare-associated infections in China provide context for the resistance ecology of these pathogens in specific healthcare settings (Yao et al., 2025). The lower hospital-wide proportions in Zigong may reflect a less ICU-enriched case mix, but antimicrobial exposure, device use, comorbidity, length of stay, colonization pressure, and molecular epidemiology were unavailable.

Among Gram-positive organisms, MRSA accounted for approximately 25% of the *S. aureus* isolates in this study, with none exhibiting resistance to vancomycin or linezolid, according to **Supplementary Table S3**. This represents a slight decrease from the 28.4% MRSA rate reported by CHINET in 2025 (Ding et al., 2026). *E. faecium* showed strong resistance to both ampicillin and fluoroquinolone antibiotics. The strongest presence of Gram-positive bacteria within the local area was due to *E. faecalis*, exhibiting resistance to linezolid at rates of 17.3% to 19.0% annually. This is substantially higher than the 5.9% linezolid resistance rate reported by CHINET for *E. faecalis* in 2025 (Ding et al., 2026). The MIC for 50% of isolates (MIC50) was 2 mg/L, while that for 90% of isolates (MIC90) was 8 mg/L. Modern genome analysis research has revealed the mechanisms by which *enterococci* develop linezolid resistance. Resistance is developed through 23S rRNA/ribosomal mutations and the presence of three transferable genetic elements: optrA, poxtA, and cfr. The optrA gene is the most common resistance element and acts upon *E. faecalis* strains that are of Chinese origin and which spread globally (Wang et al., 2015, Wang et al., 2024; Brenciani et al., 2024; Beh et al., 2025). The research in this study used gradient diffusion to confirm resistance, rather than the standard broth microdilution method. Molecular characterization was not conducted. Retesting of isolates using reference methods and conducting molecular investigations is recommended prior to drawing conclusions regarding the mechanisms of resistance, transmission links, and clonality of these bacteria (Suo et al., 2025; Martínez-Ayala et al., 2025).

The adult *H. influenzae* pattern was the clearest adjusted temporal signal. Ampicillin resistance increased from 70.0% in 2023 to 88.1% in 2025, β-lactamase positivity from 70.0% to 84.8%, and amoxicillin/clavulanate resistance from 16.0% to 59.7%; the ampicillin and amoxicillin/clavulanate associations with calendar year persisted after adjustment. CHINET 2025 reported adult *H. influenzae* β-lactamase positivity of 66.7% and ampicillin resistance of 66.2% (Ding et al., 2026). A 2026 multicenter study of lower-respiratory *H. influenzae* in neighboring Chongqing likewise reported lower overall amoxicillin/clavulanate resistance than the 2025 value observed here and identified both β-lactamase- and PBP3-associated mechanisms (Huang et al., 2026). The concurrent increase in β-lactamase positivity, MICs, and amoxicillin/clavulanate resistance is compatible with a possible contribution from non-β-lactamase mechanisms such as altered PBP3, but it does not establish such mechanisms without molecular testing. PBP3 alterations caused by *ftsI* mutations can contribute to BLNAR and β-lactamase-positive amoxicillin/clavulanate-resistant phenotypes (Dabernat et al., 2002; Denizon et al., 2024; Sfeir, 2025; Huang et al., 2026); because no molecular testing was performed here, this mechanistic explanation remains hypothetical. The adult *H. influenzae* denominator also increased substantially across study years, so changes in culture ordering, testing utilization, or case mix may have contributed to the observed calendar-year pattern; the temporal findings should therefore be interpreted as associations rather than causal secular effects.

From a clinical-practice perspective, these findings are best treated as local susceptibility evidence, not as direct evidence of treatment effect. The low CRE rate points to strong *in vitro* carbapenem activity against most *E. coli* and *K. pneumoniae* isolates in this dataset, but it does not justify indiscriminate empirical carbapenem use. CRAB and CRPA were concentrated in ICU/critical-care settings, which argues for setting-specific antibiograms, review of prior cultures and antimicrobial exposure when available, prompt organism identification and AST, and susceptibility-guided optimization or de-escalation instead of broad anti-CRAB/anti-CRPA coverage for all patients. When linezolid is being considered, unexpected *E. faecalis* linezolid non-susceptibility should trigger laboratory verification. Because adult *H. influenzae* isolates had a high *in vitro* resistance proportion in 2025, the local susceptibility pattern observed should be weighed together with regularly updated antibiograms when empirical treatment guidance is reviewed; this study does not estimate clinical treatment effectiveness. These applications are consistent with CLSI M39 principles for cumulative AST reporting (Clinical and Laboratory Standards Institute, 2022). However, patient-level therapy, recurrence, length of stay, and mortality were not linked, so treatment effectiveness and outcome effects cannot be estimated from this study.

Several limitations should be considered. First, since our retrospective, single-center surveillance mainly captured inpatient data (99.1% of isolates), these findings might not translate well to outpatient or community environments. Second, we lacked linked clinical details like patient comorbidities, previous antibiotic use, and overall outcomes. Without this information, we cannot easily separate true infection from mere colonization—which is especially tricky for CRPA and CRAB in the ICU—nor can we gauge the actual clinical burden of the resistance. Third, our phenotypic AST results were not confirmed by reference broth microdilution or molecular methods. This is particularly notable regarding the high linezolid non-susceptibility in *E. faecalis* and the growing β-lactam resistance in *H. influenzae*. Finally, time-based trends should be viewed with caution, as shifts in the patient mix, testing algorithms, or physician culture-ordering habits might partly explain the epidemiological changes we observed.

## 5 Conclusion

At this tertiary hospital in Zigong, antimicrobial resistance differed noticeably depending on patient age and care setting. Carbapenem resistance in *E. coli* and *K. pneumoniae* remained rare, while CRAB and CRPA were mostly found in ICU and critical care samples. Many *E. faecalis* isolates appeared non-susceptible to linezolid on phenotypic tests; this specific finding needs confirmation through reference testing and, if verified, further molecular analysis. Additionally, adult *H. influenzae* showed a sharp rise in resistance to ampicillin and amoxicillin/clavulanate, a trend that held true even after adjusting for patient case-mix variables. These results highlight why facilities must regularly update their cumulative antibiograms—ideally breaking them down by care setting and, sample size permitting, by age. Unexpected resistance patterns also require strict verification. Lastly, since we did not track individual antibiotic use or clinical outcomes, readers should avoid using this data to judge stewardship effectiveness or treatment success.

## Data Availability

The original contributions presented in the study are included in the article/**Supplementary Material**. Further inquiries can be directed to the corresponding author.
