# Supplementary material for: Epidemiological characteristics and antimicrobial resistance profiles of clinical bacterial isolates in a tertiary hospital in Zigong, southwestern China, 2023–2025
Source: Front Cell Infect Microbiol. 2026 Oct 14;16:1947772. doi: 10.3389/fcimb.2026.1947772 (PMC13713356; doi:10.3389/fcimb.2026.1947772)
Supplement: Supplementary file 1 [file Table1.docx]

**Supplementary Table S1** Five most frequent organisms by major specimen group.

| Specimen group | N group | Rank | Organism | n | % |
| --- | --- | --- | --- | --- | --- |
| Sputum and bronchoalveolar lavage | 7384 | 1 | *H. influenzae* | 1565 | 21.2 |
| Sputum and bronchoalveolar lavage | 7384 | 2 | *P. aeruginosa* | 1297 | 17.6 |
| Sputum and bronchoalveolar lavage | 7384 | 3 | *K. pneumoniae* | 905 | 12.3 |
| Sputum and bronchoalveolar lavage | 7384 | 4 | *M. catarrhalis* | 872 | 11.8 |
| Sputum and bronchoalveolar lavage | 7384 | 5 | *S. pneumoniae* | 820 | 11.1 |
| Urine | 3612 | 1 | *E. coli* | 2050 | 56.8 |
| Urine | 3612 | 2 | *K. pneumoniae* | 314 | 8.7 |
| Urine | 3612 | 3 | *E. faecium* | 288 | 8.0 |
| Urine | 3612 | 4 | *E. faecalis* | 265 | 7.3 |
| Urine | 3612 | 5 | *P. mirabilis* | 95 | 2.6 |
| Abdominal fluid | 2216 | 1 | *E. coli* | 1458 | 65.8 |
| Abdominal fluid | 2216 | 2 | *K. pneumoniae* | 166 | 7.5 |
| Abdominal fluid | 2216 | 3 | *P. aeruginosa* | 157 | 7.1 |
| Abdominal fluid | 2216 | 4 | *E. faecium* | 55 | 2.5 |
| Abdominal fluid | 2216 | 5 | *S. anginosus* | 49 | 2.2 |
| Pus/secretion | 2098 | 1 | *S. aureus* | 750 | 35.7 |
| Pus/secretion | 2098 | 2 | *E. coli* | 463 | 22.1 |
| Pus/secretion | 2098 | 3 | *K. pneumoniae* | 180 | 8.6 |
| Pus/secretion | 2098 | 4 | *P. aeruginosa* | 122 | 5.8 |
| Pus/secretion | 2098 | 5 | *S. agalactiae* | 64 | 3.1 |
| Blood | 1316 | 1 | *E. coli* | 683 | 51.9 |
| Blood | 1316 | 2 | *K. pneumoniae* | 157 | 11.9 |
| Blood | 1316 | 3 | *S. aureus* | 104 | 7.9 |
| Blood | 1316 | 4 | *P. aeruginosa* | 38 | 2.9 |
| Blood | 1316 | 5 | *E. faecium* | 37 | 2.8 |
