# Supplementary material for: Epidemiological characteristics and antimicrobial resistance profiles of clinical bacterial isolates in a tertiary hospital in Zigong, southwestern China, 2023–2025
Source: Front Cell Infect Microbiol. 2026 Oct 14;16:1947772. doi: 10.3389/fcimb.2026.1947772 (PMC13713356; doi:10.3389/fcimb.2026.1947772)
Supplement: Supplementary file 2 [file Table2.docx]

**Supplementary Table S2** Detailed resistance proportions for major Gram-negative organisms by year, with *P* for trend and Benjamini–Hochberg FDR q values. Each yearly cell is resistant isolates/isolates with interpretable AST (%).

| Organism | Antimicrobial | 2023 | 2024 | 2025 | *P* for trend | FDR q |
| --- | --- | --- | --- | --- | --- | --- |
| E. coli | Piperacillin/tazobactam | 43/1590 (2.7%) | 29/1544 (1.9%) | 38/1714 (2.2%) | 0.361 | 0.972 |
| E. coli | Ceftriaxone | 642/1590 (40.4%) | 653/1544 (42.3%) | 727/1714 (42.4%) | 0.240 | 0.960 |
| E. coli | Ceftazidime | 226/1590 (14.2%) | 230/1544 (14.9%) | 238/1714 (13.9%) | 0.775 | 0.972 |
| E. coli | Cefepime | 235/1590 (14.8%) | 239/1544 (15.5%) | 249/1714 (14.6%) | 0.826 | 0.972 |
| E. coli | Ertapenem | 21/1590 (1.3%) | 19/1544 (1.2%) | 26/1714 (1.5%) | 0.972 | 0.972 |
| E. coli | Imipenem | 16/1590 (1.0%) | 17/1544 (1.1%) | 21/1714 (1.2%) | 0.548 | 0.972 |
| E. coli | Meropenem | 17/1590 (1.1%) | 15/1544 (1.0%) | 21/1714 (1.2%) | 0.657 | 0.972 |
| E. coli | Amikacin | 32/1590 (2.0%) | 42/1544 (2.7%) | 51/1714 (3.0%) | 0.082 | 0.492 |
| E. coli | Gentamicin | 563/1590 (35.4%) | 513/1544 (33.2%) | 547/1714 (31.9%) | 0.034 | 0.408 |
| E. coli | Ciprofloxacin | 900/1590 (56.6%) | 868/1544 (56.2%) | 966/1714 (56.4%) | 0.892 | 0.972 |
| E. coli | Levofloxacin | 779/1590 (49.0%) | 769/1544 (49.8%) | 853/1714 (49.8%) | 0.661 | 0.972 |
| E. coli | Trimethoprim/sulfamethoxazole | 758/1590 (47.7%) | 766/1544 (49.6%) | 830/1714 (48.4%) | 0.682 | 0.972 |
| K. pneumoniae | Piperacillin/tazobactam | 36/496 (7.3%) | 42/592 (7.1%) | 45/681 (6.6%) | 0.655 | 0.866 |
| K. pneumoniae | Ceftriaxone | 85/496 (17.1%) | 116/592 (19.6%) | 109/681 (16.0%) | 0.515 | 0.866 |
| K. pneumoniae | Ceftazidime | 52/496 (10.5%) | 73/592 (12.3%) | 63/681 (9.3%) | 0.404 | 0.866 |
| K. pneumoniae | Cefepime | 56/496 (11.3%) | 76/592 (12.8%) | 68/681 (10.0%) | 0.408 | 0.866 |
| K. pneumoniae | Ertapenem | 21/496 (4.2%) | 13/592 (2.2%) | 25/681 (3.7%) | 0.722 | 0.866 |
| K. pneumoniae | Imipenem | 16/496 (3.2%) | 12/592 (2.0%) | 22/681 (3.2%) | 0.891 | 0.955 |
| K. pneumoniae | Meropenem | 19/496 (3.8%) | 11/592 (1.9%) | 22/681 (3.2%) | 0.671 | 0.866 |
| K. pneumoniae | Amikacin | 10/496 (2.0%) | 11/592 (1.9%) | 25/681 (3.7%) | 0.060 | 0.720 |
| K. pneumoniae | Gentamicin | 50/496 (10.0%) | 71/592 (12.0%) | 62/681 (9.1%) | 0.491 | 0.866 |
| K. pneumoniae | Ciprofloxacin | 120/496 (24.2%) | 141/592 (23.8%) | 166/681 (24.4%) | 0.926 | 0.955 |
| K. pneumoniae | Levofloxacin | 79/496 (16.0%) | 83/592 (14.0%) | 95/681 (14.0%) | 0.364 | 0.866 |
| K. pneumoniae | Trimethoprim/sulfamethoxazole | 108/496 (21.8%) | 140/592 (23.6%) | 137/681 (20.1%) | 0.422 | 0.866 |
| P. aeruginosa | Piperacillin/tazobactam | 65/439 (14.8%) | 78/547 (14.3%) | 108/673 (16.0%) | 0.694 | 0.776 |
| P. aeruginosa | Ceftazidime | 58/439 (13.2%) | 63/547 (11.5%) | 88/673 (13.1%) | 0.638 | 0.758 |
| P. aeruginosa | Cefepime | 36/439 (8.2%) | 44/547 (8.0%) | 56/673 (8.3%) | 0.982 | 0.982 |
| P. aeruginosa | Aztreonam | 53/439 (12.1%) | 50/547 (9.1%) | 42/673 (6.2%) | 0.003 | 0.038 |
| P. aeruginosa | Imipenem | 31/439 (7.1%) | 52/547 (9.5%) | 71/673 (10.5%) | 0.157 | 0.258 |
| P. aeruginosa | Meropenem | 19/439 (4.3%) | 27/547 (4.9%) | 33/673 (4.9%) | 0.879 | 0.928 |
| P. aeruginosa | Ciprofloxacin | 71/439 (16.2%) | 109/547 (19.9%) | 105/673 (15.6%) | 0.116 | 0.258 |
| P. aeruginosa | Levofloxacin | 58/439 (13.2%) | 90/547 (16.5%) | 98/673 (14.6%) | 0.340 | 0.431 |
| P. aeruginosa | Tobramycin | 16/439 (3.6%) | 22/547 (4.0%) | 12/673 (1.8%) | 0.056 | 0.258 |
| A. baumannii | Ceftazidime | 53/140 (37.9%) | 39/121 (32.2%) | 33/119 (27.7%) | 0.215 | 0.292 |
| A. baumannii | Cefepime | 54/140 (38.6%) | 37/121 (30.6%) | 34/119 (28.6%) | 0.188 | 0.275 |
| A. baumannii | Imipenem | 53/140 (37.9%) | 38/121 (31.4%) | 32/119 (26.9%) | 0.163 | 0.258 |
| A. baumannii | Meropenem | 53/140 (37.9%) | 38/121 (31.4%) | 32/119 (26.9%) | 0.163 | 0.258 |
| A. baumannii | Amikacin | 50/140 (35.7%) | 36/121 (29.8%) | 29/119 (24.4%) | 0.142 | 0.258 |
| A. baumannii | Gentamicin | 50/140 (35.7%) | 34/121 (28.1%) | 27/119 (22.7%) | 0.074 | 0.258 |
| A. baumannii | Tobramycin | 48/140 (34.3%) | 38/121 (31.4%) | 28/119 (23.5%) | 0.154 | 0.258 |
| A. baumannii | Ciprofloxacin | 54/140 (38.6%) | 38/121 (31.4%) | 33/119 (27.7%) | 0.162 | 0.258 |
| A. baumannii | Levofloxacin | 50/140 (35.7%) | 26/121 (21.5%) | 23/119 (19.3%) | 0.004 | 0.038 |
| A. baumannii | Trimethoprim/sulfamethoxazole | 33/140 (23.6%) | 34/121 (28.1%) | 18/119 (15.1%) | 0.049 | 0.258 |
