# Supplementary material for: Epidemiological characteristics and antimicrobial resistance profiles of clinical bacterial isolates in a tertiary hospital in Zigong, southwestern China, 2023–2025
Source: Front Cell Infect Microbiol. 2026 Oct 14;16:1947772. doi: 10.3389/fcimb.2026.1947772 (PMC13713356; doi:10.3389/fcimb.2026.1947772)
Supplement: Supplementary file 3 [file Table3.docx]

**Supplementary Table S3** Detailed resistance proportions for major Gram-positive organisms and H. influenzae by year, with *P* for trend and Benjamini–Hochberg FDR q values. Each yearly cell is resistant isolates/isolates with interpretable AST (%).

| Organism | Antimicrobial | 2023 | 2024 | 2025 | *P* for trend | FDR q |
| --- | --- | --- | --- | --- | --- | --- |
| S. aureus | Oxacillin (MRSA phenotype) | 116/466 (24.9%) | 105/458 (22.9%) | 119/510 (23.3%) | 0.575 | 1.000 |
| S. aureus | Gentamicin | 40/466 (8.6%) | 43/458 (9.4%) | 47/510 (9.2%) | 0.726 | 1.000 |
| S. aureus | Ciprofloxacin | 54/466 (11.6%) | 63/458 (13.8%) | 71/510 (13.9%) | 0.280 | 0.687 |
| S. aureus | Levofloxacin | 53/466 (11.4%) | 62/458 (13.5%) | 71/510 (13.9%) | 0.238 | 0.687 |
| S. aureus | Clindamycin | 109/466 (23.4%) | 146/458 (31.9%) | 124/510 (24.3%) | 0.795 | 1.000 |
| S. aureus | Erythromycin | 258/466 (55.4%) | 279/458 (60.9%) | 265/510 (52.0%) | 0.271 | 0.687 |
| S. aureus | Linezolid | 0/466 (0%) | 0/458 (0%) | 0/510 (0%) | 1.000 | 1.000 |
| S. aureus | Vancomycin | 0/466 (0%) | 0/458 (0%) | 0/510 (0%) | 1.000 | 1.000 |
| S. aureus | Tetracycline | 76/466 (16.3%) | 84/458 (18.3%) | 98/510 (19.2%) | 0.234 | 0.687 |
| E. faecium | Ampicillin | 111/125 (88.8%) | 117/124 (94.4%) | 152/160 (95.0%) | 0.096 | 0.518 |
| E. faecium | Ciprofloxacin | 105/125 (84.0%) | 107/124 (86.3%) | 133/160 (83.1%) | 0.757 | 1.000 |
| E. faecium | Levofloxacin | 105/125 (84.0%) | 106/124 (85.5%) | 133/160 (83.1%) | 0.860 | 1.000 |
| E. faecium | Linezolid non-susceptibility | 5/125 (4.0%) | 4/124 (3.2%) | 9/160 (5.6%) | 0.599 | 1.000 |
| E. faecium | Vancomycin | 3/125 (2.4%) | 3/124 (2.4%) | 5/160 (3.1%) | 0.912 | 1.000 |
| E. faecium | Tetracycline | 45/125 (36.0%) | 57/124 (46.0%) | 75/160 (46.9%) | 0.140 | 0.630 |
| E. faecalis | Ampicillin | 2/98 (2.0%) | 1/100 (1.0%) | 2/127 (1.6%) | 0.847 | 1.000 |
| E. faecalis | Ciprofloxacin | 39/98 (39.8%) | 45/100 (45.0%) | 49/127 (38.7%) | 0.607 | 1.000 |
| E. faecalis | Levofloxacin | 38/98 (38.8%) | 44/100 (44.0%) | 48/127 (37.9%) | 0.619 | 1.000 |
| E. faecalis | Linezolid non-susceptibility | 17/98 (17.3%) | 19/100 (19.0%) | 24/127 (18.9%) | 0.777 | 1.000 |
| E. faecalis | Vancomycin | 0/98 (0%) | 0/100 (0%) | 0/127 (0%) | 1.000 | 1.000 |
| E. faecalis | Tetracycline | 89/98 (90.8%) | 88/100 (88.0%) | 100/127 (78.7%) | 0.026 | 0.209 |
| H. influenzae | Ampicillin | 217/262 (82.8%) | 500/594 (84.2%) | 623/711 (87.6%) | 0.031 | 0.209 |
| H. influenzae | Amoxicillin/clavulanate | 65/262 (24.8%) | 221/594 (37.2%) | 385/711 (54.1%) | <0.001 | 0.027 |
| H. influenzae | Cefuroxime | 164/262 (62.6%) | 407/594 (68.5%) | 473/711 (66.5%) | 0.652 | 1.000 |
| H. influenzae | Trimethoprim/sulfamethoxazole | 172/262 (65.6%) | 367/594 (61.8%) | 488/711 (68.6%) | 0.190 | 0.687 |
| H. influenzae | Chloramphenicol | 9/262 (3.4%) | 24/594 (4.0%) | 46/711 (6.5%) | 0.026 | 0.209 |
| H. influenzae | Tetracycline | 14/262 (5.3%) | 18/594 (3.0%) | 28/711 (3.9%) | 0.542 | 1.000 |
